# The regulation of immune checkpoints by the hypoxic tumor microenvironment

**DOI:** 10.7717/peerj.11306

**Published:** 2021-05-07

**Authors:** Min Hu, Yongfu Li, Yuting Lu, Miao Wang, Yingrui Li, Chaoying Wang, Qin Li, Hong Zhao

**Affiliations:** 1 Department of Biochemistry & Molecular Biology, Basic Medical College, Shanxi Medical University, Taiyuan, Shanxi Province, China; 2 Department of Oncology, Beijing Friendship Hospital, Capital Medical University, Beijing, China; 3 Department of Oncology, The Second Affiliated Hospital of Hainan Medical University, Haikou, Hainan Province, China

**Keywords:** Tumor microenvironment, Hypoxia, Immune checkpoints, Immune escape, Drug treatment

## Abstract

The tumor microenvironment (TME) influences the occurrence and progression of tumors, and hypoxia is an important characteristic of the TME. The expression of programmed death 1 (PD1)/programmed death-ligand 1 (PDL1), cytotoxic T-lymphocyte-associated antigen 4 (CTLA4), and other immune checkpoints in hypoxic malignant tumors is often significantly increased, and is associated with poor prognosis. The application of immune checkpoint inhibitors (ICIs) for treating lung cancer, urothelial carcinoma, and gynecological tumors has achieved encouraging efficacy; however, the rate of efficacy of ICI single-drug treatment is only about 20%. In the present review, we discuss the possible mechanisms by which the hypoxic TME regulates immune checkpoints. By activating hypoxia-inducible factor-1*α* (HIF-1*α*), regulating the adenosine (Ado)-A2aR pathway, regulating the glycolytic pathway, and driving epithelial-mesenchymal transition (EMT) and other biological pathways, hypoxia regulates the expression levels of CTLA4, PD1, PDL1, CD47, lymphocyte activation gene 3 (LAG3), T-cell immunoglobulin and mucin domain 3 (TIM3), and other immune checkpoints, which interfere with the immune effector cell anti-tumor response and provide convenient conditions for tumors to escape immune surveillance. The combination of HIF-1*α* inhibitors, Ado-inhibiting tumor immune microenvironment regulatory drugs, and other drugs with ICIs has good efficacy in both preclinical studies and phase I-II clinical studies. Exploring the effects of TME hypoxia on the expression of immune checkpoints and the function of infiltrating immune cells has greatly clarified the relationship between the hypoxic TME and immune escape, which is of great significance for the development of new drugs and the search for predictive markers of the efficacy of immunotherapy for treating malignant tumors. In the future, combination therapy with hypoxia pathway inhibitors and ICIs may be an effective anti-tumor treatment strategy.

## Introduction

The expression of programmed death 1 (PD1) / programmed death-ligand 1 (PDL1), cytotoxic T-lymphocyte-associated antigen 4 (CTLA4), and other immune checkpoints, is often significantly increased in malignant tumors and is associated with poor prognosis (Liu et al., 2020a; Ma et al., 2019). Immune checkpoint inhibitors (ICIs) have shown encouraging efficacy in lung cancer, urothelial carcinoma, and gynecologic tumors (Hamanishi et al., 2015; Rittmeyer et al., 2017; Sharma et al., 2017). However, the efficacy rate of ICI monotherapy is only approximately 20% (Nanda et al., 2016). Therefore, the factors influencing immune checkpoint expression in tumors and how the efficacy of ICIs can be improved are of urgent concern.

The tumor microenvironment (TME) influences the occurrence and progression of tumors. The components of the TME are complex, including malignant tumor cells, immune cells, inflammatory cells, endothelial cells, cancer-associated fibroblasts (CAFs), and extracellular matrix. Different types of stromal cells and extracellular matrixes constitute different microenvironments, which can be roughly divided into the hypoxic microenvironment, immune microenvironment, stromal microenvironment, and angiogenesis microenvironment. Hypoxia is one of the characteristics of the TME, and its effects on tumor immunosuppression and immune escape are attracting increasing attention. Previous studies have shown that tumor hypoxia can lead to the exhaustion of T cells and natural killer (NK) cells (Chambers & Matosevic, 2019; Liu et al., 2020b). In addition, hypoxia can promote the recruitment of immunosuppressive cells, such as regulatory T cells (Tregs) and tumor-related macrophages (TAMs), as well as the expression of immunosuppressive molecules, such as vascular endothelial growth factor (VEGF), transforming growth factor-β (TGF-β), and interleukin-10 (IL-10), thereby preventing immune effector cells from exerting their anti-tumor roles (Terry, Buart & Chouaib, 2017). Hypoxia can also regulate the expression levels of CTLA4, PD1, PDL1, CD47, lymphocyte activation gene 3 (LAG3), T-cell immunoglobulin and mucin domain 3 (TIM3), as well as other immune checkpoints, through a variety of mechanisms to inhibit T cell activation, thus inhibiting the body’s anti-tumor immune response, leading to tumor immune escape. In the present review, we discuss the mechanisms by which the hypoxic microenvironment affects immune checkpoint expression, and the progress towards treatment regimens that resist these mechanisms, including combination ICI therapies, for the treatment of malignant tumors.

## Survey Methodology

To ensure a thorough literature search, we searched 4,612,862 articles using the keywords “tumor”, “hypoxia”, “HIF-1*α*”, “TME”, “immune checkpoint”, “PD1”, “PDL1”, “CTLA4”, “CD47”, “adenosine”, “glycolysis”, and “EMT” in the PubMed database. As a result, 130 articles that were related to tumors, hypoxia, or immune checkpoints were included in the present review.

### The relationship between hypoxia and tumor

#### The effect of hypoxia on the TME

The interaction between tumor cells and tumor stromal cells creates a unique TME. Infinite proliferation is the main malignant phenotype of tumor cells, and is also the basis of tumor invasion and metastasis. However, the rapid proliferation of tumor cells is challenged by external factors. The rapid growth of tumor cells is an important cause of TME hypoxia, and is one of the characteristic features of the TME (Hiraga, 2018). Hypoxia has a wide range of effects on the behavior of tumor cells and changes to the TME. In the TME, cells are exposed to hypoxia and increased acidity, so tumor cells must aggressively regulate the intra- and extracellular pH by secreting angiogenic factors that stimulate vascular endothelial cell proliferation and migration, inducing angiogenesis, which in turn supplies adequate blood and oxygen to tumor cells, thus creating the ideal conditions for the invasion of the surrounding tissue. Next, the response of tumor cells to hypoxia increases the stability of the hypoxia-inducible factor-1 (HIF-1) transcription complex, which in turn promotes angiogenesis (Palazon et al., 2017), anaerobic metabolism, and tumor cell growth and invasion. In addition, hypoxia also promotes the weakening of the adhesion of epithelial-derived tumor cells to stromal cells by activating the epithelial-mesenchymal transition (EMT), which induces the invasion and migration of tumor cells (Yoshimoto et al., 2019). Finally, hypoxia is one of the driving factors of anti-tumor immunosuppression in the TME (Park et al., 2019). Hypoxia can stimulate the expression of immunosuppressive molecules such as VEGF, adenosine (Ado), and TGF-β within the TME (Munn & Jain, 2019). It can also hinder the differentiation and maturation of dendritic cells (DCs) (Munn & Jain, 2019), drive TAMs from M1 to M2 (Yin et al., 2019), as well as recruit Treg cells (Wu et al., 2019), myeloid-derived suppressor cells (MDSCs) (Guo et al., 2018) and other immunosuppressive cells. As a result, tumor antigen (Ag) presentation and T lymphocyte activation and proliferation are limited, leading to anti-tumor immunosuppression. The hypoxic microenvironment therefore plays an important role in promoting the progression and metastasis of tumors, and suppressing anti-tumor immunity.

#### The effects of hypoxia on tumor cell death

Hypoxia has both inhibitory and promoting effects on the regulation of tumor cell death through mediating the autophagy of tumor cells. On one hand, autophagy increases the immunogenicity of tumor cells through the chronic release of damage-associated molecular patterns (DAMPs), maturation of dendritic cells (DCs) and release of immunomodulatory molecules. As a result, autophagy stimulates the body’s immune response to tumor and kills tumor cells, namely immunogenic cell death (ICD) (Garg et al., 2015) . Michael R. Olin et al. used 5% O2 and atmospheric oxygen (20% O2) separately to culture GL261 glioma cells and EMT6 breast cancer cells. The results showed that the lysates of 5% O2 cultured tumor cells can significantly prolong the survival time of mice glioma and breast cancer model. The main mechanism is to enhance the antigen-presenting ability of DCs and the cross-presentation of exogenous antigens on MHC-I, and to strengthen the proliferation and killing ability of Ag-specific T cells (Olin et al., 2011; Olin et al., 2010). On the other hand, in the hypoxic TME, autophagy can produce enough ATP to promote the growth of tumor cells through free amino acids and free fatty acids, thus it helps tumor cells overcome necrosis and apoptosis, leading to drug resistance of tumor. Autophage can also induce the polarization of macrophages to M2 type in the TME and reduce the infiltration of cytotoxic T cells and natural killer (NK) cells, leading to immune escape of tumor cells and suppression of anti-tumor immunity (Zaarour et al., 2021). Therefore, the regulation of hypoxia-induced autophagy on tumor cell death is complex and contradictory (Vito, El-Sayes & Mossman, 2020).

### Tumor immune checkpoints

In the TME, tolerogenic DCs, non-functional T cells, and Treg cells can induce immunosuppression and affect the anti-tumor immune response (Jafari et al., 2020). The negative costimulatory molecules known as immune checkpoints play an important role in T cells. Common immune checkpoints include CTLA4, PD1, PDL1, LAG3, and TIM3, which negatively regulate the immune response at different developmental stages of various tumor types (Andrews et al., 2017; Bertucci & Goncalves, 2017; Burugu et al., 2018; Joller & Kuchroo, 2017; Zhao & Subramanian, 2017). ICIs are targets for immunotherapy, which is a revolutionary breakthrough in cancer treatment (Mpekris et al., 2020). Anti-CTLA4 antibodies (Abs) and anti-PD1/PDL1 Abs can suppress immune checkpoints and thereby enhance the anti-tumor immune response of immune cells. These antibodies have been approved by the FDA for the treatment of melanoma, urothelial carcinoma, and renal cell carcinoma (RCC) (Hargadon, Johnson & Williams, 2018). Currently, immunotherapy is the standard therapy for treating many different cancers (Munn & Jain, 2019; Xin Yu, Hubbard-Lucey & Tang, 2019). Here, we will discuss the individual immune checkpoints with available Abs that are currently used as part of immunotherapy regimens, and their effect on the TME.

#### PD1/PDL1

PD1, also known as cluster of differentiation 279 (CD279), is an important immunosuppressive molecule that is mainly expressed on activated T cells, B cells, DCs, NK cells, and monocytes (Chen et al., 2016). PDL1, also known as cluster of differentiation 274 (*CD274*) or B7 homolog 1 (B7-H1), is a protein encoded by the *CD274* gene in humans, and is widely expressed on the surface of tumor cells. Under normal circumstances, the immune system responds to foreign agents that accumulate in the lymph nodes or spleen, triggering Ag-specific CD8(+) cytotoxic T lymphocyte proliferation. The binding of PD1 and PDL1 can inhibit the proliferation of CD8(+) T cells in lymph nodes, and inhibit autoimmune diseases and transplant rejection (Kuol et al., 2018). However, in the TME, PDL1 on the surface of tumor cells interacts with PD1 on the surface of activated CD8(+) T cells, which not only inhibits the proliferation and differentiation of T cells, but also interferes with the function of T cells, leading to functional unresponsiveness, exhaustion, and apoptosis of T cells. The PD1/PDL1 pathway is one of the important mechanisms leading to tumor immune escape. Blocking the PD1/PDL1 signaling pathway can enhance the immune surveillance and killing ability of T cells to tumor cells, and activate the tumor immune response, and has therefore been used in cancer immunotherapies. Complete or partial response to a wide range of malignancies has been achieved with anti-PD1 Abs such as nivolumab and pembrolizumab, and anti-PDL1 Abs such as atezolizumab, durvalumab, and avelumab, including lung cancer (Antonia et al., 2018), melanoma (Eggermont et al., 2018), and urothelial carcinoma (Farina, Lundgren & Bellmunt, 2017). Anti-PD1/PDL1 Abs have been widely used in clinical practice as a new approach to cancer treatment, and are excellent candidates for combination therapy.

#### CTLA4

CTLA4, also known as CD152, is a member of the immunoglobulin superfamily and is expressed by activated T cells. CTLA4 is a transmembrane receptor that primarily transmits inhibitory signals to T cells. CTLA4 was first discovered by Golstein et al. in 1987. In November 1995, Sharpe et al. discovered the function of CTLA4 as a negative regulator of T cell activation by knocking out CTLA4 in mice. CTLA4 and CD28 are homologous receptors, and they compete with the ligands CD80 (B7-1) and CD86 (B7-2), respectively, which are expressed on the surface of antigen-presenting cells (APCs), to deliver inhibitory or activation signals to T cells. The main function of CD28 is to form a dimer with the ligand CD80, together with the CD86 monomer, to activate the second signal of T cell activation, and to stimulate the activation of T cells in coordination with the first signal of TCR-mediated T cell activation. However, when a specific Ag activates lymphoid tissue, CTLA4 expression is up-regulated on the surface of activated naive and memory T cells (Chen et al., 2016). CTLA4 also interacts with CD80 and CD86 with a higher affinity and avidity than CD28 (Rowshanravan & Halliday, 2018), thus inhibiting the CD28 mediated second signal of T cell activation and obstructing the immune response of T cells. In the physiological state, CTLA4 acts as a negative regulator of T cell activation, and plays a role in inducing immune tolerance to auto-antigens and protecting normal cells from T cell attack (Hosseini et al., 2020; Salama, Adbeltawab & Tayebi, 2019). However, tumor cells inhibit T cell proliferation and differentiation by inducing the expression of CTLA4 on the T cell–surface to bind to the B7 molecules on the APC, which inactivates T cells infiltrating tumor tissue, leading to immune escape of tumor cells. By blocking the binding of CTLA4 and B7, anti-CTLA4 Abs can activate the function of tumor-infiltrating T cells and achieve anti-tumor immunotherapy. Anti-CTLA4 antibodies such as ipilimumab and tremelimumab are effective for treating melanoma (Larkin et al., 2019; Sharma et al., 2020) and prostate cancer (Slovin et al., 2013). Combination therapy with anti-PD1 and anti-CTLA4 inhibitors has become an important approach to cancer treatment in the field of checkpoint inhibition. Clinical studies have shown that a combination of anti-PD1 and anti-CTLA4 Abs is more effective in treating multiple cancer types than either Ab alone. Anti-CTLA4 therapy enhances the antigen-specific T cell-dependent immune response, while anti-PD1/PDL1 activates the ability of CD8(+) T cells to lyse cancer cells, resulting in mutually beneficial synergistic effects (Wei et al., 2019).

#### LAG3

LAG3 belongs to the immunoglobulin (Ig) superfamily, and is mainly expressed in human activated T cells and NK cells. It has four extracellular Ig-like domains (D1-D4) that are highly homologous to CD4. CD4 is mainly expressed on T helper (Th) cells, and are receptors for Th cell antigen recognition, which, when combined with the peptide section of the major histocompatibility complex class II (MHC-II), participate in the identification of Ags by Th cells. After naive CD4(+) T cells receive Ags stimulation, they can differentiate into T cells of different subtypes under different conditions and perform different functions. As a negative regulator of the immune response, LAG3 has an affinity for MHC-II that is 100 times stronger than that of CD4 molecules (Hosseini et al., 2020; Salama, Adbeltawab & Tayebi, 2019), and negatively regulates the function of T cells. Fibrinogen-like protein 1 (FGL1) is another functional ligand of LAG3, which is expressed in large amounts on the surface of cancer cells, and can inhibit the activation of Ag-specific T cells, leading to the immune escape of tumors.

In addition, LAG3 is expressed on Treg cells, and can promote Treg cell differentiation and inhibit the activity of cytotoxic T cells (Turnis et al., 2016). The number of CD8(+) T cells was significantly increased in mice with LAG3 deficiency compared to in mice with LAG3 expression (Workman & Vignali, 2005). Blocking LAG3/FGL1 and PD1/PDL1 pathways simultaneously may play a synergistic role in antitumor therapy (Wang et al., 2019a). At present, clinical studies on the safety, tolerance, and efficacy of specific anti-LAG3 Abs (such as BMS 986016, LAG525, and MK-4280) for the treatment of various malignant tumors with a single drug or combined with anti-PD1 inhibitors are underway (Joller & Kuchroo, 2017). An anti-LAG3 monoclonal antibody (mAb) is expected to be a promising antitumor immunotherapy drug.

#### TIM3

TIM3 is an immunomodulatory protein member of the TIM family, and an immune checkpoint. In addition to being expressed on tumor cells, it is also expressed on a variety of immune cells, such as CD4(+)CD8(+) T cells that produce interferon (IFN) (Ohmura et al., 2020), Treg cells (Fu et al., 2019), DCs, NK cells (Sarhan et al., 2018), myeloid cells (Wang et al., 2019b) and mast cells (Phong et al., 2015). TIM3 is thought to be a “co-inhibitory” or “checkpoint” receptor, and is expressed in dysfunctional or exhausted T cells in chronic viral infection and cancers (Wolf, Anderson & Kuchroo, 2020). In the TME, TIM3 mainly mediates T cell apoptosis and participates in tumor immunosuppression, and occurrence and development of tumors. TIM3 has four types of ligands, including carcinoembryonic antigen-related cell adhesion molecule 1 (CEACAM1), high-mobility group box 1 (HMGB1), phosphatidylserine (PtdSer), and Galectin-9 (Gal-9) (Tao et al., 2020). Gal-9 was the first ligand to be identified, and its binding with TIM3 inhibits the T cell immune response and induces tumorigenesis.Tumor growth rates were significantly increased in TIM3-overexpressing EL4 mice (Nakajima et al., 2019). In a mouse head and neck tumor model, blocking TIM3 could decrease the number of TIM3(+) Treg cells and slow down the growth rate of tumors (Liu et al., 2018). In solid tumors, such as gastric cancer, non-small cell lung cancer (NSCLC), and colon cancer, high expression of TIM3 is associated with poor prognosis (Zhang et al., 2017). In addition, TIM3 is positively correlated with PD1 expression in CD8(+) T cells, and the secretion of interleukin-2 (IL-2), interferon-γ (IFN-γ), and tumor necrosis factor (TNF) by TIM3(+)PD1(+)CD8(+) T cells was decreased (Sakuishi et al., 2010). Preclinical studies have found that blocking TIM3 and PD1 simultaneously can enhance the immune response of T cells and cause tumor regression (Wolf, Anderson & Kuchroo, 2020). Currently, clinical trials on the efficacy of TIM3 inhibitors such as TSR-022, LY3321367, MGB453, and INCAGN02390, as a single drug or in combination with PD1 inhibitors, in malignant tumors are underway (Ahn, 2018; Harding et al., 2019; Klein et al., 2019; Murtaza et al., 2016; Waight et al., 2018). TIM3 is expected to be an important target for anti-tumor immunotherapy.

#### CD47

CD47 is a membrane protein that is widely expressed in the body in normal tissue, such as red blood cells, mainly as a self-protective protein. The corresponding ligand of CD47 is signal regulatory protein-α (SIRP-α), which is mainly expressed on macrophages. When it binds to CD47, it transmits inhibitory signals and inhibits the phagocytic activity of macrophages. CD47-SIRP-α interaction-induced suppression signals can inhibit macrophages from attacking normal tissue, and thus protect healthy cells from being destroyed by the immune system. CD47 also exists on the surface of cancer cells, where it is more abundant than on normal cells. Tumor cells use the mechanism of action between CD47 and macrophages to escape phagocytosis, causing the body to lose its first line of defense against tumor cells (Noman et al., 2018). Drug development for CD47 targets is currently underway (Ma et al., 2020).

### Possible mechanisms by which the hypoxicTME affects immune checkpoint expression

Hypoxia is a driving factor for immunosuppression in the TME. The hypoxic microenvironment may affect the expression levels of immune checkpoints by activating HIF-1α, regulating the Ado-A2a receptor (A2aR) axis, regulating glycolysis, and driving EMT. Hypoxia-associated molecules lead to the up- or down-regulation of co-inhibitory immune checkpoints (Table 1), which affects the anti-tumor effects of immune effector cells. At the same time, hypoxia can cause ICIs to be ineffective (Jayaprakash et al., 2018). Hypoxia is one of the mechanisms by which malignant tumors evade immune surveillance. Here, we will discuss the mechanisms by which the hypoxic TME affects the expression of immune checkpoints, and the possible implications for the use of ICIs immunotherapy.

**Table 1:** The regulation of immune checkpoints by hypoxia-associated molecules.

**Hypoxia-associated molecules**	**Immune checkpoints**	**Impact on immune checkpoints**	**References**
HIF-1α	PDL1	Stimulatory	Lequeux et al. (2019) and Zhou et al. (2019)
HIF-1α	CTLA4, LAG3	Stimulatory	Doedens et al. (2013)
PKM2, PHD3	PDL1	Stimulatory	Palsson-McDermott et al. (2017)
CA9	PDL1	Stimulatory	Giatromanolaki & Harris (2020)
Ado, A2aR, A2bR, cAMP	PD1, CTLA4	Stimulatory	Hatfield et al. (2015)
Ado	PD1	Stimulatory	Allard et al. (2013)
Lactate, GPR81, TAZ, TEAD	PDL1	Stimulatory	Feng et al. (2017)
LDH-A	PDL1	Stimulatory	Seth et al. (2017)
ZEB1	PDL1	Stimulatory	Chen et al. (2014)
SNAIL	PDL1	Stimulatory	Noman et al. (2017)
ZEB1, SNAIL	CD47	Stimulatory	Noman et al. (2018)
cAMP, PKA	PDL1	Inhibitory	Feng et al. (2017)
miR-200	PDL1	Inhibitory	Noman et al. (2017)

#### Relationship between HIF-1α activation and immune checkpoint expression in solid tumors

**Figure 1:**
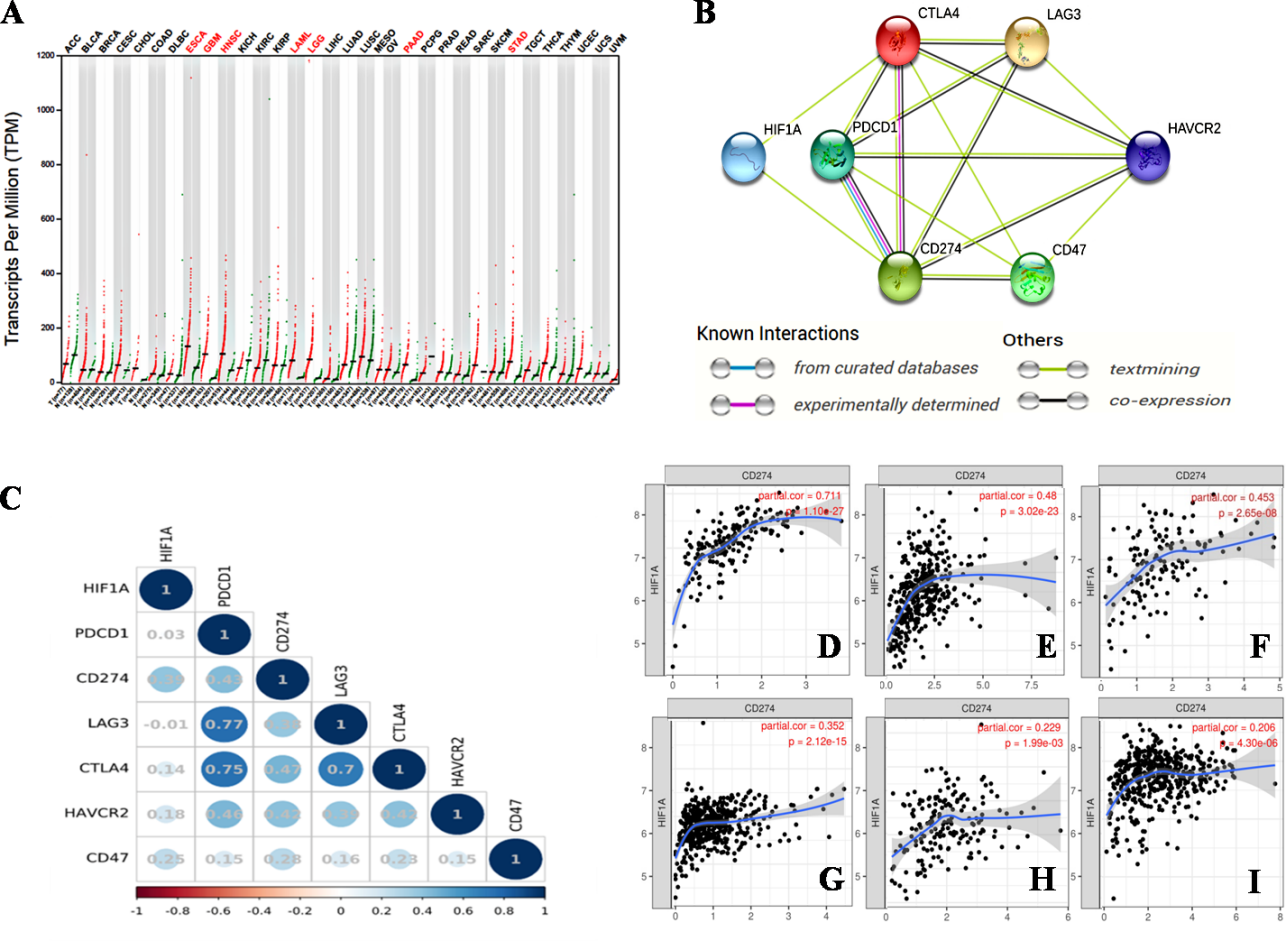
The relationship between HIF-1α and immune checkpoints. (A) *HIF1A* expression in various tumors. *HIF1A* is overexpressed in seven types of tumors of ESCA, GBM, HNSC, LAML, LGG, PAAD and STAD. (B) The protein-proteininteraction (PPI) network constructed by *HIF1A, CD274, PDCD1, CTLA4, LAG3, CD47* and *HAVCR2* genes. HIF-1α is directly related to PDL1 and CTLA4, according to the analysis of the literature. (C) The correlation between *HIF1A* and immunotherapy-related target genes in carcinomas. The upper abscissa and left ordinate represent *HIF1A* and immune checkpoint genes, and the lower abscissa represents Pearson’s correlation coefficient (r). The maximum diameter of circle represents the size of absolute r value in the circle. Blue and red circles with different shades represent different degrees of positive and negative correlation, respectively. (D) The correlation between *HIF1A* and *CD274* expression in PAAD. (E) The correlation between *HIF1A* and *CD274* expression in STAD. (F) The correlation between *HIF1A* and *CD274* expression in GBM. (G) The correlation between *HIF1A* and *CD274* expression in LGG. (H) The correlation between *HIF1A* and *CD274* expression in ESCA. (I) The correlation between *HIF1A* and *CD274* expression in HNSC. The abscissa represents the expression levels of *CD274* and the ordinate represents the expression levels of *HIF1A*. Image credit: (A) GEPIA web at http://gepia.cancer-pku.cn/detail.php. (B) STRING web at https://string-db.org/. (C) Bioinformastics at http://www.bioinformatics.com.cn/. (D–I) TIMER at https://cistrome.shinyapps.io/timer/. Abbreviations: HIF-1α, Hypoxia-inducible factor-1 *α*; ESCA, Esophageal cancer; GBM, Glioblastomamultiforme; HNSC, Head and neck squamous cell carcinoma; LGG, Lower grade glioma; STAD, Stomachadenocarcinoma; CTLA4, Cytotoxic T-lymphocyte-associated antigen 4; LAG3, Lymphocyte activation gene 3; PDL1, Programmed death-ligand 1; LAML, Acute myeloid leukemia.

Hypoxia is an important characteristic of the TME, and plays an important role in the occurrence and progression of tumors. The activation of HIF-1 creates conditions for tumor cells to adapt to a continuous hypoxic microenvironment (Balamurugan, 2016). HIF-1 is a nuclear transcription factor (TF). As a member of the HIF family, HIF-1 affects tumor growth by regulating a variety of signaling molecules and TFs, such as VEGF, endothelin-1 (ET-1), and glycolytic enzymes (Balamurugan, 2016). HIF-1 α is an oxygen-dependent subunit of HIF-1 that forms the heterodimer structure of HIF-1 with another constitutively expressed protein, the HIF-1β subunit (Li et al., 2016). The activation of HIF-1α promotes tumor angiogenesis (Du et al., 2019), promotes distant metastasis of tumor cells (Balamurugan, 2016), mediates aerobic glycolysis in tumor cells (Chen et al., 2018b), and decreases the pH of the TME (Balamurugan, 2016). Kitajima et al. have shown that the disease-specific survival (DSS) of patients with gastric cancer expressing HIF-1 is significantly shorter than that of patients without HIF-1 expression (Kitajima & Miyazaki, 2013). Jin et al. (2016) found that the combined expression of HIF-1α and carbonic anhydrase 9 (CA9) protein was a prognostic factor for significantly shortened disease-free survival (DFS) in patients with triple-negative breast cancer (TNBC). Thus, HIF-1α is a poor prognostic indicator for various malignancies (Swartz, Wegner & Noorlag, 2020). We searched the GEPIA database on 33 types of malignancies and found that the *HIF1A* gene was overexpressed in esophageal cancer (ESCA), glioblastomamultiforme (GBM), head and neck squamous cell carcinoma (HNSC), acute myeloid leukemia (LAML), lower grade glioma (LGG), pancreatic adenocarcinoma (PAAD), and stomach adenocarcinoma (STAD) (Fig. 1A).

HIF-1α is a transcriptional activator of *CD274*. By combining hypoxia response element 1 (HRE1) and hypoxia response element 4 (HRE4) of the proximal promoter of PDL1, HIF-1α induces PDL1 up-regulationin tumor cells and immune cells, including MDSCs, macrophages, DCs, and bone marrow-derived macrophages (BMDMs) in the TME (Barsoum et al., 2014; Lequeux et al., 2019; Noman et al., 2014). The *HIF1A* gene of follicular thyroid cancer (FTC) cells is knocked out, which down-regulates PDL1 expression on the surface of tumor cells and inhibits tumor cell growth (Zhou et al., 2019).

**Figure 2:**
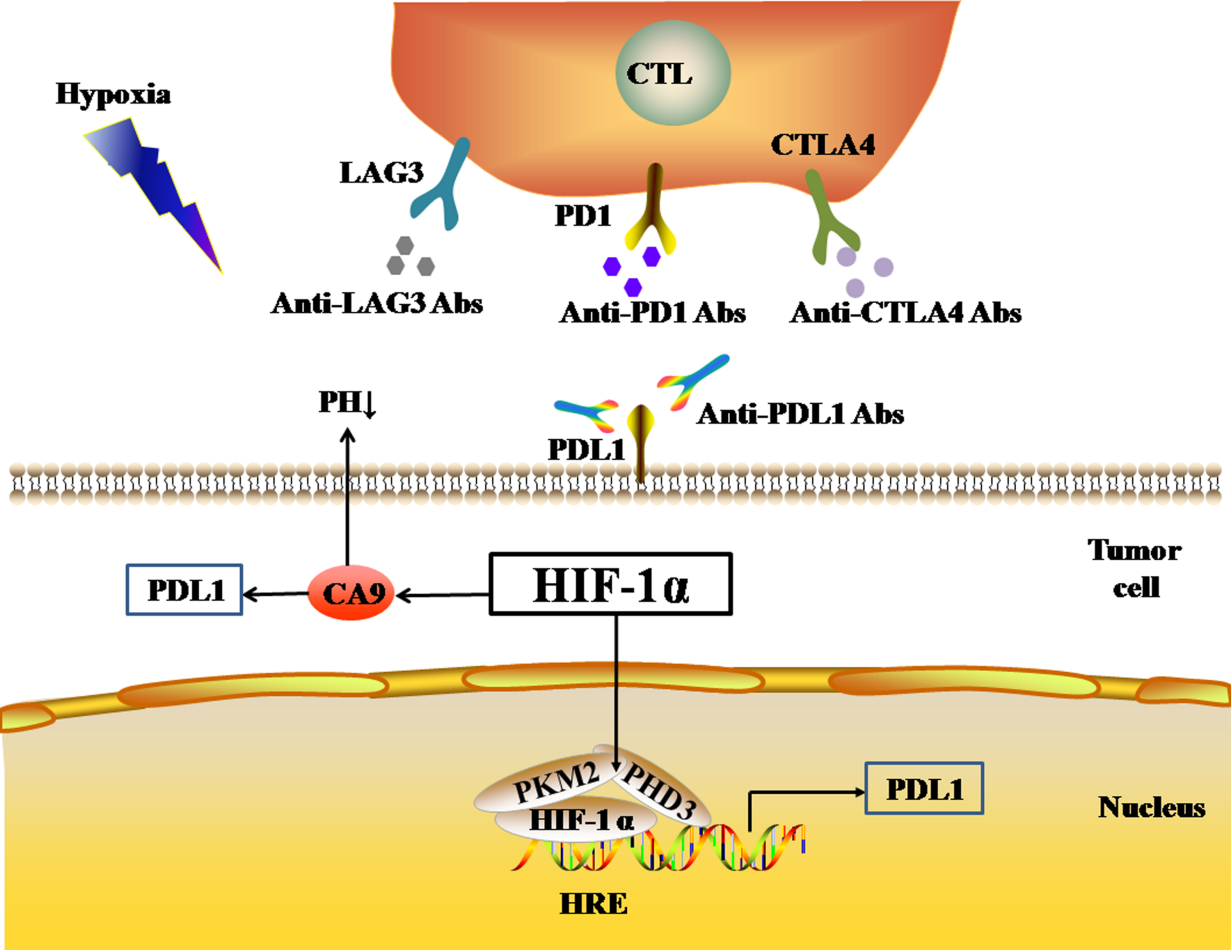
Activated HIF-1α regulates the expression levels of immune checkpoints. The black arrows represent promotion in the figure. As a nuclear transcription factor, HIF-1α can bind with PKM2 and PHD3 forming a complex and bind to the HRE site on PDL1 promoter to up-regulate the expression of PDL1 in tumor cells. HIF-1α can also regulate the expression of PDL1 by activating CA9. Abbreviations: HIF-1α, Hypoxia-inducible factor-1α; PKM2, Pyruvate kinase-M2; HRE, Hypoxia response element; PHD3, A co-activator; PDL1, Programmed death-ligand 1; CA9: Carbonic anhydrase 9.

Pyruvate kinase-M2 (PKM2) is an important regulator in the process of aerobic glycolysis (the Warburg effect) of tumor cells, catalyzing the final rate-limiting step of glycolysis. PKM2 often switches between tetramer and dimer forms to determine whether glucose is converted to pyruvate for energy supply or biosynthesis. PKM2 is usually present as a dimer in tumor cells. Palsson-McDermott et al. found that the dimerized PKM2, as the PHD3-dependent co-activator of HIF-1, can translocate to the nucleus, bind to HIF-1, and bind to the HRE site on the PDL1 promoter to up-regulate the expression of PDL1 in macrophages, DCs, T cells, and tumor cells (Palsson-McDermott et al., 2017) (Fig. 2). The PKM2 activator TEPP-46 can tetramerize PKM2, and thus down-regulate the expression of PDL1 in macrophages, DCs, T cells, and tumor cells by inhibiting the binding of PKM2 and HIF-1 complexes to the HRE site on the PDL1 promoter (Palsson-McDermott et al., 2017).

We identified a correlation between HIF-1α and PDL1 using the STRING database (https://string-db.org/) (Fig. 1B), and found a significant positive correlation between HIF-1α and PDL1 expression in cancer using the UCSC Xena database (https://xena.ucsc.edu/) (*r* = 0.393, *P* < 0.001) (Fig. 1C). According to the TIMER database (https://cistrome.shinyapps.io/timer/), HIF-1α is significantly positively correlated with PDL1 expression in PAAD (Fig. 1D), STAD (Fig. 1E), GBM (Fig. 1F), LGG (Fig. 1G), ESCA (Fig. 1H), HNSC (Fig. 1I), and especially in PAAD, STAD, and GBM (*r* = 0.453 − 0.711, *P* < 0.001). Thus, the activation of HIF-1α promotes PDL1 expression in a variety of solid tumors.

In addition, HIF-1 can also up-regulate the expression of PDL1 in tumor cells through the activation of CA9. CA9 causes tissue acidosis by hydrating CO_2_ and releasing bicarbonate and protons into the extracellular environment. Tissue acidosis (pH 6.0 ∼7.0) is a prominent feature of solid tumors. Solid tumors, such as breast cancer, brain tumors, sarcomas, and malignant melanomas, usually have a pH ranging 5.7–7.0. Low pH can inhibit the function of tumor-infiltrating immune cells, such as the cytotoxic function of CD8(+) T cells and the IFN production function of Th1 cells (Erra Díaz, Dantas & Geffner, 2018). Giatromanolaki & Harris (2020) observed higher expression levels of PDL1 in tumor cells overexpressing CA9 in vitro. HIF-1 is an important factor that promotes the activation of CA9 in tumor cells in a hypoxic microenvironment. HIF-1 can activate CA9 and up-regulate the expression of PDL1 in tumor cells, thereby inhibiting the anti-tumor function of CD8+ T cells. In addition to its effect on the expression of PDL1, HIF-1α can also up-regulate the expression of CTLA4 and LAG3 in CD8(+) T cells (Doedens et al., 2013). However, the protein-protein interaction (PPI) networks accessed via the STRING database only showed an interaction between HIF-1 α and CTLA4, but no interaction between HIF-1α and LAG3 (Fig. 1B). The TIMER database showed that *HIF1A* expression was very low or not correlated with the expression of CTLA4 and LAG3, respectively.

#### The Ado-A2aR pathway promotes immune checkpoint expression

**Figure 3:**
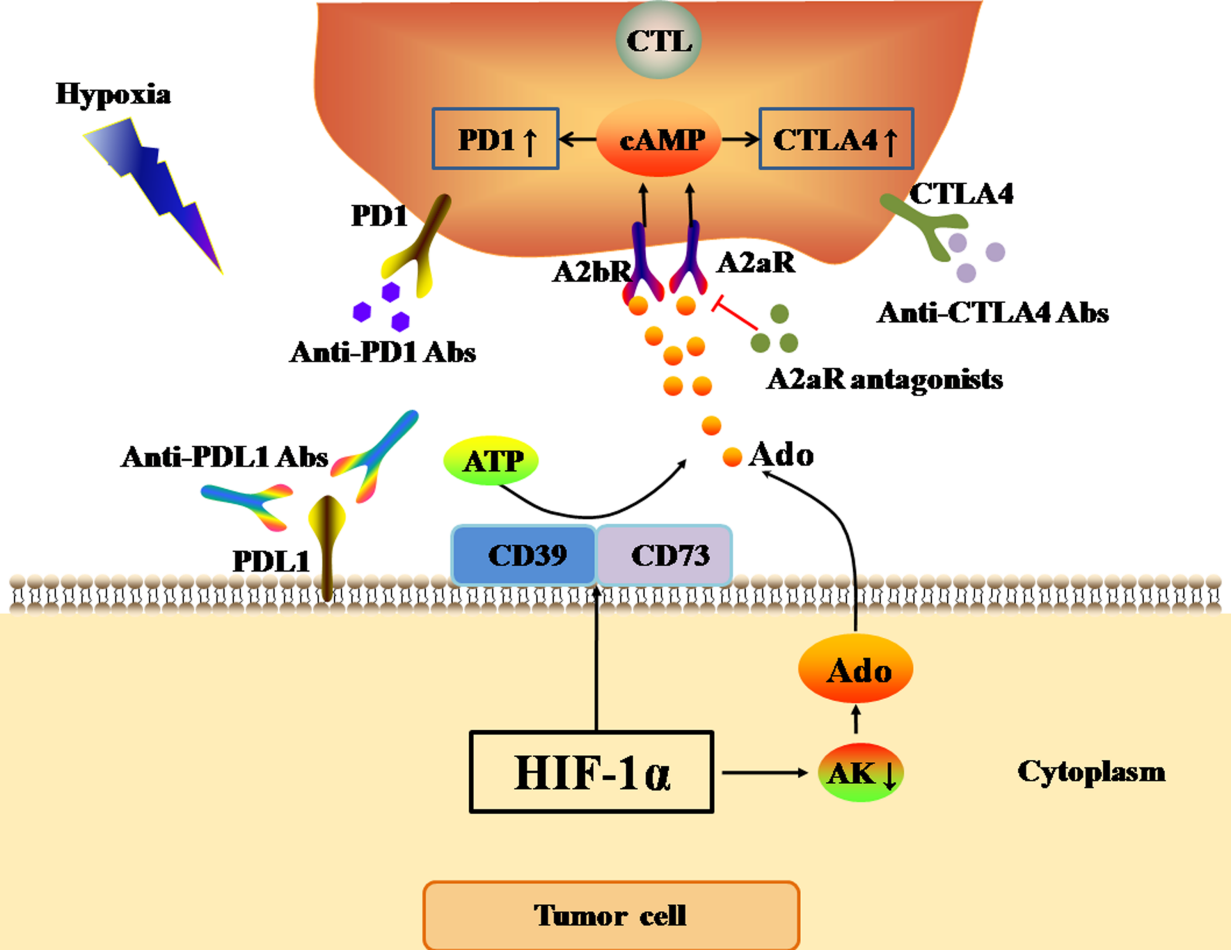
Ado-A2aR pathway regulates the expression levels of immune checkpoints. The black and red arrows represent promotion and inhibition, respectively. The accumulation of Ado in TME is mainly promotes by HIF-1α through two ways. One way is up-regulating the expression of CD39 and CD73 to cleaved ATP into Ado. The other way is inhibiting the activity of AK. AK activation can catalyze the conversion of Ado to AMP, which is the main Ado clearance pathway. Finally, Ado activates Ado-A2aR pathway to up-regulate the expression of PD1 and CTLA4 in CTLs in tumor cells. Abbreviations: Ado, Adenosine; A2aR, A2a receptor; HIF-1α, Hypoxia-inducible factor-1α; AK, Adenosine kinase; ATP, Adenosine triphosphate; PD1, Programmed death 1; CTLA4, Cytotoxic T-lymphocyte-associated antigen 4; CTL, Cytotoxic T cell.

One of the most important features of the hypoxic TME is the accumulation of Ado in the stroma (Ohta et al., 2006). In normal tissue, the hypoxia-Ado-A2aR pathway mainly delivers immunosuppressive signals, and thus protects normal tissue from damage caused by the autoimmune response and inflammation. However, in the TME, the hypoxia-Ado-A2aR pathway plays a protective role for cancer cells. The activation of HIF-1 α in tumors can up-regulate the expression of CD73 and CD39 on the cell surface under hypoxia (Young et al., 2014). Adenosine triphosphate (ATP) released from blood in microvessels in the TME is first cleaved into adenosine monophosphate (AMP) by CD39 on the tumor cell surface. Subsequently, AMP is further cleaved to Ado by CD73 (Ghiringhelli et al., 2012). Adenosine kinase (AK) is a cytosolic enzyme that catalyzes the conversion of Ado to AMP, and its activation represents the main Ado clearance pathway. However, Ado accumulation in the TME and activated HIF-1α inhibit AK activity (Leone, Horton & Powell, 2015), further exacerbating Ado accumulation in the hypoxic TME. The accumulation of Ado, in combination with the A2aR and A2b receptor (A2bR) on the surface of anti-tumor T cells, promotes the expression of cyclic adenosine monophosphate (cAMP) in anti-tumor T cells. Consequently, Ado accumulation up-regulates the expression of PD1, CTLA4, and TGF-β on the surface of T cells, and inhibits the secretion of IFN, leading to the functional inhibition of anti-tumor T cells (Hatfield et al., 2015; Leone, Horton & Powell, 2015) (Fig. 3). However, Allard et al. found that Ado only up-regulated the expression of PD1 on tumor-infiltrated CD8(+) T cells and CD4(+) T cells, but did not affect the expression of CTLA4 (Allard et al., 2013). In conclusion, the hypoxia-Ado-A2aR pathway can promote the immune escape of tumor cells, and lead to tumor progression and metastasis by up-regulating the expression of PD1 and other inhibitory immune checkpoints on the surface of anti-tumor T cells.

#### Hypoxia regulates glycolysis

**Figure 4:**
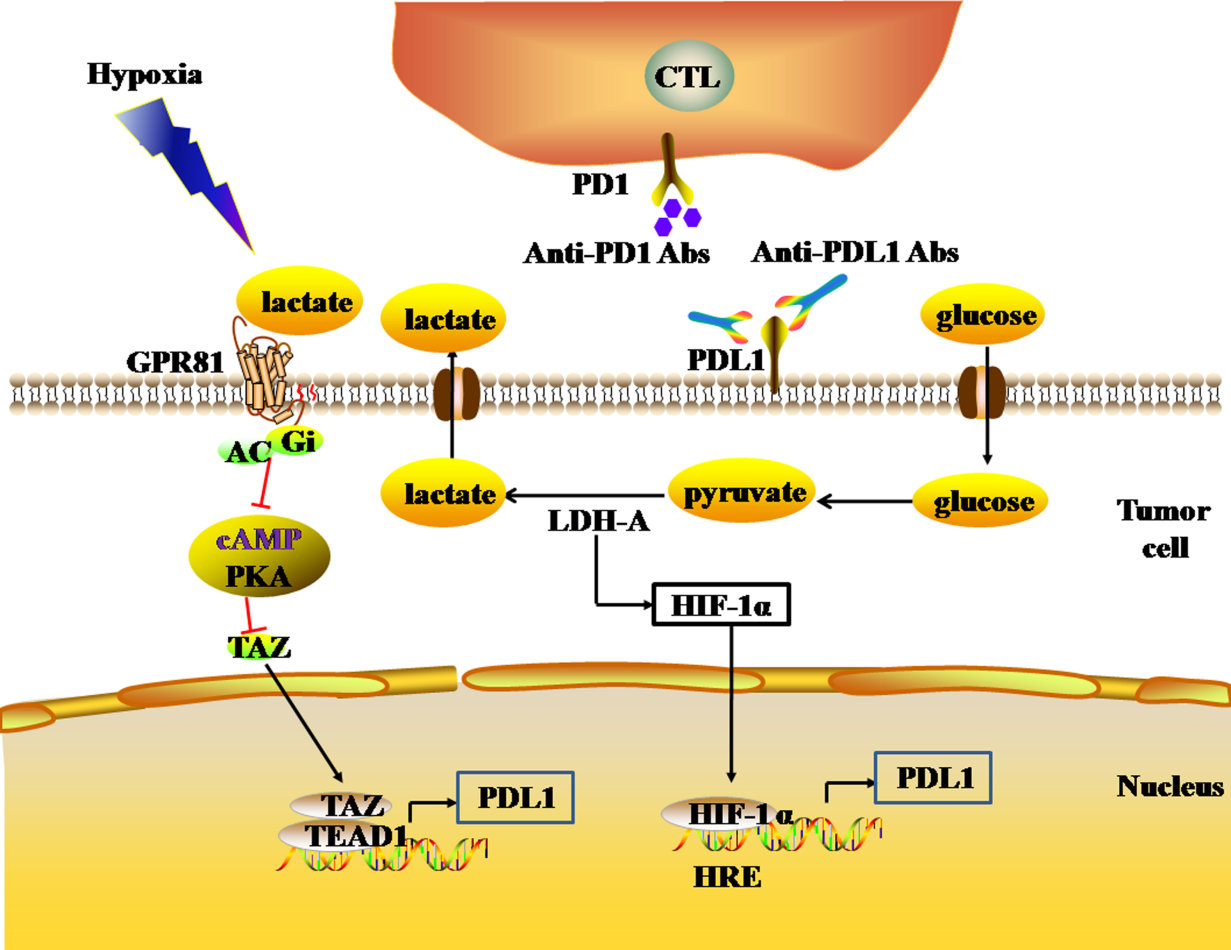
The glycolytic pathway regulates the expression levels of immune checkpoints. The black and red arrows represent promotion and inhibition, respectively. The expression of HIF-1α is up-regulated by LDH-A. HIF-1α can combine to HRE to promote the expression of PDL1. LDH-A also catalyze pyruvate to produce large amounts of lactate. The accumulation of lactate actives GPR81-cAMP-PKA pathway to up-regulate the expression of PDL1 in tumor cells. Abbreviations: LDH-A, Lactate dehydrogenase A; HIF-1 α, Hypoxia-inducible factor-1α; PDL1, Programmed death-ligand 1; GPR81, G protein-coupled receptor 81; cAMP, Cyclic adenosine monophosphate; PKA, Protein kinase A.

Glycolysis is the main mechanism of energy metabolism in tumor cells. During the aerobic oxidation of glucose, one molecule of glucose can produce 36 or 38 ATP molecules. Glycolysis, by contrast, is an inefficient method of energy metabolism, producing only two molecules of ATP per molecule of glucose. Due to the deficiency of energy metabolism caused by mitochondrial respiratory injury of tumor cells, the energy metabolism of tumor cells under aerobic conditions is aerobic glycolysis (the Warburg effect), which is similar to the energy metabolism mode of tumor cells under hypoxia. During glycolysis, lactate dehydrogenase (LDH) catalyzes pyruvate to produce large amounts of lactic acid, and tumor cells produce large amounts of lactic acid, forming an acidic TME. The high LDH expression in the TME reflects the active glycolysis metabolism of tumor cells, and is associated with poor prognosis in melanoma, RCC, prostate cancer, and other solid tumors (Petrelli et al., 2015; Van Wilpe et al., 2020). In the acidic TME, lactic acid activates G protein-coupled receptor 81 (GPR81) by binding with GPR81, which inhibits the generation of cAMP and the activity of protein kinase A (PKA) in tumor cells, and then activates the downstream transcriptional co-activator TAZ. TAZ forms a complex with the TF (TEAD), which induces up-regulation of PDL1 expression in tumor cells, thereby inhibiting the anti-tumor effect of infiltrating T lymphocytes (Feng et al., 2017) (Fig. 4). In addition, lactate dehydrogenase A (LDH-A) in the acidic TME can induce the expression of HIF-1α. LDH-A is a subunit that constitutes a homotetrameric or heterotetrameric LDH enzyme. LDH-A deficiency inhibits the expression of HIF-1α in tumor cells (Serganova et al., 2018). Since HIF-1α is the transcriptional activator of the PDL1 gene, the loss of LDH-A leads to a decrease in the number of tumor cells expressing PDL1, and an increase in the number of infiltrating T cells. As a result, the intratumoral anti-tumor immune response increases (Seth et al., 2017) (Fig. 4). Thus, there is a close relationship between the glycolysis of tumor cells and the expression of PDL1.

#### Hypoxia drives the EMT

**Figure 5:**
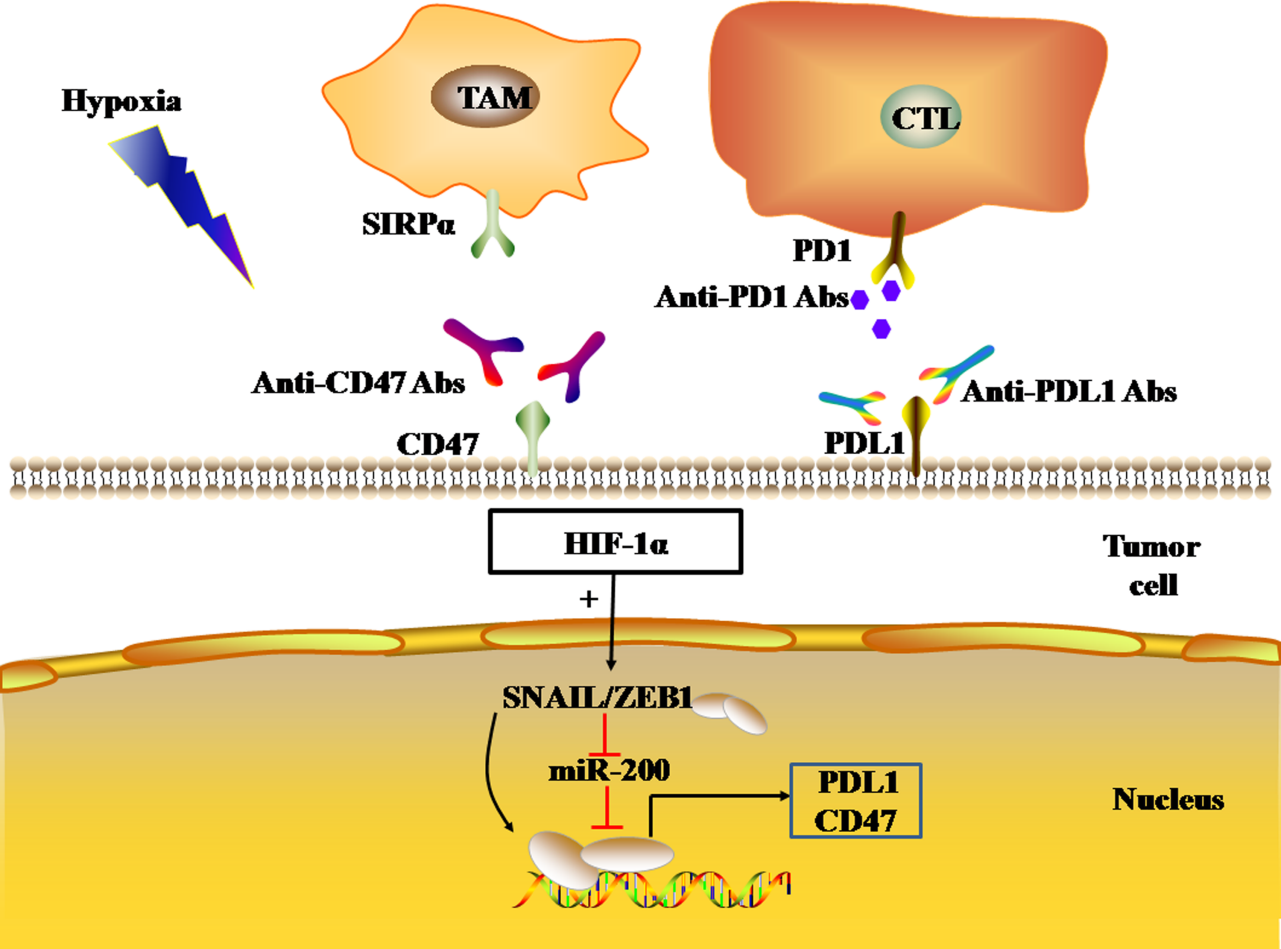
EMT regulates the expression levels of immune checkpoints. The black and red arrows represent promotion and inhibition, respectively. HIF-1α promotes EMT-induced gene transcription and regulates the expression of PDL1 and CD47 in tumor cells. Abbreviations: EMT, Epithelial-mesenchymal transition; HIF-1α, Hypoxia-inducible factor-1α; PDL1, Programmed death-ligand 1.

EMT is the process by which epithelial cells lose their polarity and transform into the mesenchymal phenotype, resulting in cell invasion and metastasis, including induction of stem cell properties, reduction of apoptosis, and promotion of immunosuppression. EMT plays a key role in tumor cell metastasis, and the loss of E-cadherin expression is a key step in the EMT. E-cadherin inhibitors are known as EMT-induced genes, and can be divided into two groups according to their effects: genes that bind to the promoter of E-cadherin and inhibit its expression, and genes that indirectly inhibit the activity of E-cadherin. The former includes SNAIL, ZEB, E47, and KLF8, while the latter includes TWIST, goosecoid, E2.2, and FOXC2. EMT regulation is a complex network that includes HIF signaling pathways associated with hypoxia. Hypoxia-induced activation of HIFs activates EMT-related signaling pathways and drives the occurrence of EMT by regulating EMT-related TFs (Joseph et al., 2018). ZEB1, a member of the ZEB family, inhibits E-cadherin expression, resulting in EMT. HIF-1 can combine the HRE in the proximal promoters of ZEB1, SNAIL, and TWIST, increase the expression of ZEB1, SNAIL, and TWIST, and thus down-regulate the expression of E-cadherin in tumor cells, leading to EMT (Yang et al., 2008; Zhang et al., 2015; Zhu et al., 2013; Zhu et al., 2018). Using a bioinformatics approach, M. P. Mak et al. analyzed the gene pathways related to EMT characteristics in the datasets of 11 types of malignant tumors, including adenocarcinoma, lung cancer, colon cancer, ovarian cancer, and bladder cancer, and showed that high EMT scores were significantly correlated with the high expression of PDL1, PD1, and CTLA4 et al. immune checkpoints (Mak et al., 2016). Chen, L. and M. Z. Noman et al. found that the transcriptional activation of the EMT-induced gene ZEB1 can up-regulate the expression of PDL1 on the surface of tumor cells, which is related to miR-200. MiR-200 is the main regulatory molecule of EMT, which not only plays a role in maintaining the epithelial phenotype, but also inhibits the expression of PDL1 by targeting *CD274* genes. ZEB1 up-regulates the expression of PDL1 on the tumor cell surface by inhibiting the transcription of miR-200, thus leading to the inhibition of CD8(+) T cell function (Chen et al., 2014; Noman et al., 2017). In MDA-MB-231 and MCF7-2101 cancer cell lines, SNAIL up-regulated ZEB1 expression by binding to the ZEB1 promoter, thereby increasing the expression of PDL1 on the tumor cell surface, weakening the tumor-killing effect of cytotoxic T cells (CTLs). As a result, SNAIL and ZEB1 lead to the immune escape of tumor cells (Noman et al., 2017) (Fig. 5).

In addition, the transcriptional activation of EMT-induced genes *ZEB1* and *SNAIL1* can also induce the expression of *CD47* in tumor cells. CD47, an immune checkpoint, binds to the SIRP-α ligand to inhibit macrophage phagocytosis. In human breast cancer cells, *ZEB1* and *SNAIL1* up-regulated the expression of CD47 on the surface of tumor cells by directly binding to E-box-2 and E-box-3 promoters at the proximal end of *CD47* in tumor cells (Noman et al., 2018). In mesenchymal MDA-MB-231 breast cancer cells, the *ZEB1* or *SNAL1* gene was targeted by siRNA to down-regulate the expression of CD47 on the tumor cell surface, thus achieving the restoration of function of macrophages (Noman et al., 2018).

#### Hypoxia affects the efficacy of antitumor physical therapy

Ionizing radiation (IR), photodynamic therapy (PDT) and cold atmosphere plasma (CAP) belong to physical therapies for malignant tumors. The effectiveness of these three treatments is closely related to the oxygen content of tumor. The killing effect of low linear energy transfer (LET) on tumor cells mainly depends on the indirect effect of free radicals. As oxygen has a strong affinity with electrons, oxygen can capture ionized electrons of biomolecules, inhibit the recovery process, and fix ionizing radiation damage to biomolecules, achieving the purpose of killing tumor cells. The sensitivity of malignant tumor cells to radiotherapy under normal oxygen condition is about 2.5 times that under hypoxia condition (Hong et al., 2016). PDT plays its role mainly through the generation of singlet oxygen. PDT motivates the photosensitizer absorbed by tumor tissue and transfers energy to the surrounding oxygen, resulting in the formation of highly reactive singlet oxygen. The oxidation reaction occurs between singlet oxygen and adjacent biological macromolecule, causing cytotoxicity. The effectiveness of CAP is also associated with active oxygen (Gjika et al., 2018). Hence, hypoxia is an important factor affecting the efficacy of antitumor physical therapy.

Antitumor physical therapy can cause the death of tumor cells and the release of tumor antigens, which up-regulates the expression of MHC-I complex on tumor cells and promotes the maturation of antigen-presenting cells. Subsequently, the activation and proliferation of effector T cells are initiated by antigen-presenting cells, thus exerting anti-tumor immune response (Brodin, Guha & Tomé, 2015; Chen et al., 2020; Cramer et al., 2020; Eckert et al., 2019). However, studies have shown that radiotherapy and PDT can up-regulate the expression of PDL1 in tumor, weakening the anti-tumor immune response (Deng et al., 2014; O’Shaughnessy et al., 2018). As previously mentioned, PDL1, PD1, CTLA4, LAG3 et al. immune checkpoints are up-regulated in hypoxic tumor, thus hypoxic tumor still has a high recurrence rate after antitumor physicotherapeutics. Studies have shown that immunotherapy by blocking immune checkpoint signaling pathway can improve the efficacy of antitumor physical therapy for hypoxic tumors (Antonia et al., 2018; Chen et al., 2020; O’Shaughnessy et al., 2018).

### Progression to drug therapy

#### Ado-inhibiting tumor immune microenvironment regulatory drugs

Ado-inhibiting tumor immune microenvironment regulatory drugs are mainly based on the CD39-CD73-A2aR pathway. Inhibition of the three targets in this pathway can down-regulate Ado production and thus inhibit the immune escape of tumor cells. Combined with ICIs, such as anti-PD(L)1 or anti-CTLA4 mAbs, Ado inhibition can enhance the immune response to anti-tumor therapy (Table 2).

**Table 2:** Partial clinical trials of inhibitors targeting hypoxic pathway.

**Molecule**	**Study ID**	**Indications**	**Phase**	**Combination partner**	**Efficacy**	**References**
**A2aR antagonist**						
AZD4635	NCT03980821	Solid tumors	1	—	—	
AZD4635	NCT03381274	NSCLC	1b/2	MEDI9447	—	
AZD4635	NCT02740985	mCRPC	1	—(*n* = 4)	25% PR	Bendell et al. (2019)
				Durvalumab (*n* = 4)	25% PR, 25%CR, 50% ORR	
CPI-444	NCT02655822	RCC	1/1b	—(*n* = 33)	3.0% PR, 17% SD, mPFS 4.1m	Fong et al. (2020)
				Atezolizumab (*n* = 35)	11.4% PR, 39% SD, mPFS 5.8 m	
**A2aR/A2bR antagonist**						
AB928	NCT03720678	GEC, CRC	1/1b	mFOLFOX	—	
AB928	NCT03629756	NSCLC, breast cancer, melanoma	1	Zimberelimab	—	
**CD73 inhibitor**						
MEDI9447	NCT03616886	TNBC	1/2	Paclitaxel+carboplatin +durvalumab	—	
MEDI9447	NCT04668300	Metastatic sarcoma	2	Durvalumab	—	
AB680	NCT04104672	mPDAC	1/1b	Zimberelimab (*n* = 17)	41% ORR	Manji et al. (2021)
**CD39 inhibitor**						
SRF617	]NCT04336098	Solid Tumors	1	—	—	
RO7070179	NCT02564614	HCC	1b	—	—	
**HIF-1α inhibitor**						
BAY87-2243	NCT01297530	Advanced malignancies	1	—	—	
**CA9 inhibitor**						
SLC-0111	NCT03450018	mPDAC	1b	Gemcitabine	—	
Girentuximab	NCT00087022	ccRCC	3	—	No statistically significant DFS or OS advantage	Chamie et al. (2017)
**Glycolytic inhibitor**						
DCA	]NCT00540176	GBM	2	—	—	
**EMT inhibitor**						
AB-16B5	NCT04364620	NSCLC	2	Docetaxel	—	
AB-16B5	NCT02412462	Solid Tumors	1	—	—	

Deletion of the *A2aR* gene or antagonistic A2 receptor can activate anti-tumor T cell activity in mice, and enhance the anti-tumor immune effect, thereby inhibiting tumor growth (Kjaergaard & Hatfield, 2018; Leone, Lo & Powell, 2015b). Blocking A2aR can also significantly down-regulate immune checkpoint expression in mice, and thus enhance the therapeutic effects of ICIs (Leone, Horton & Powell, 2015). Leone et al. found that the A2aR inhibitor CPI-444 significantly reduced the expression of PD1 and LAG3 in Treg cells and CD8(+) T effector cells in tumor-bearing mice, and enhanced the function of tumor-infiltrating CD8(+) T cells (Leone et al., 2018). Based on these findings, A2a antagonists combined with ICIs may enhance the anti-tumor immune effect. The combination of A2aR antagonists and anti-PD1/CTLA4/TIM3 mAbs significantly reduced the load transfer in a mouse melanoma model, and significantly prolonged the life of the mice compared with any monotherapy (Leone, Lo & Powell, 2015). An A2a antagonist (SYN115) combined with anti-PD1 mAbs can up-regulate the expression of IFN *γ* and granzyme B in tumor-infiltrating CD8+T cells, and enhance anti-tumor immunity against CD73(+) tumors (Beavis et al., 2015). Some studies have shown that the combination of A2a antagonists and ICIs is only effective in tumor cells with a high expression of CD73, suggesting that CD73 may be a predictor of the efficacy of the combination therapy (Mittal et al., 2014). In addition to A2aR, blocking CD73 can also prevent tumor growth and metastasis (Qin et al., 2014). CD73 inhibitors have been shown to enhance the activity of anti-PD1 and anti-CTLA4 mAbs by improving the tumor immune response (Allard et al., 2013; Iannone et al., 2014).

At present, multiple phase I/II clinical trials of A2aR antagonists combined with anti-PD1/PDL1 mAbs have been carried out in advanced solid tumors (Fong et al., 2020; Leone & Emens, 2018) (Table 2). The phase I clinical trial of the A2aR antagonist AZD4635 (NCT02740985) (Bendell et al., 2019) showed that, of the eight patients with metastatic castration-resistant prostate cancer (mCRPC) with evaluable tumor lesions, one patient who received AZD4635 monotherapy achieved a partial response (PR), while one patient who received AZD4635 combined with durvalumab achieved a complete response (CR), and one patient achieved a PR. Among the four mCRPC patients with measurable tumor lesions, one patient presented with persistent prostate-specific antigen (PSA) reduction after AZD4635 monotherapy. Another phase I clinical trial was conducted on the A2aR antagonist CPI-444 (NCT02655822) (Fong et al., 2020) in 68 patients with renal cell carcinoma, including 33 patients who received CPI-444 monotherapy, and the remainder receiving CPI-444 and atezolizumab (an anti-PDL1 mAb) combination therapy. In the CPI-444 monotherapy group, one patient achieved a PR (3.0%), 17% achieved stable disease (SD) that lasted for more than 6 months, and the median PFS (mPFS) was 4.1 months. In the combination group, four patients achieved a PR (11.4%), 39% achieved SD that lasted for at least 6 months, and the mPFS was 5.8 months. Some studies have shown that the synergistic effect of A2aR antagonists with ICIs may reduce the dosage of each drug, thus reducing the occurrence of immune-related adverse events (irAEs) (Leone, Lo & Powell, 2015).

In addition, phase I/II clinical trials of CD73 inhibitors combined with anti-PD1 / PDL1 mAbs have also been carried out in a variety of solid tumors *(*Table 2*)*. Preliminary results of the phase I/Ib clinical trial ARC-8 show that the combination of the CD73 inhibitor AB680 and anti-PD1 mAbs for the treatment of metastatic pancreatic cancer can obtain an objective response rate (ORR) of 41% (Manji et al., 2021). In summary, the current research on A2aR and CD73 target antagonists is still in the preliminary stage, but initial efficacy has been demonstrated. The CD73-Ado-A2aR pathway may be a new target for immunotherapy.

#### HIF-1α inhibitors

Glyceryl trinitrate (GTN), a nitric oxide signaling agonist, can inhibit the accumulation of HIF-1α in anoxic cells, and down-regulate the expression of PDL1, thus enhancing the ability of cytotoxic T cells (CTLs) to lyse tumor cells (Barsoum et al., 2014). Similarly, Xing et al. confirmed, both *in vivo* and in vitro, that fraxinellone can reduce the nuclear accumulation of HIF-1α in A549 cells during hypoxia, and reduce the expression of PDL1, thus enhancing the anti-tumor immune function of the body (Xing et al., 2018). PX-478 is a selective HIF-1α inhibitor. Both the knockdown of PX-478 and HIF-1α can down-regulate the expression of PDL1 in ESCC cells (Zhu et al., 2017). In conclusion, HIF-1α inhibitors have a synergistic effect with PDL1 inhibitors, and the combined application of these inhibitors may improve their individual antitumor therapeutic effect (Dai et al., 2018). At present, the development of HIF-1α inhibitors for monotherapy of a variety of solid tumors is still in phase I of clinical trials (Table 2), and clinical results have not yet been published.

#### Glycolytic pathway or CA9 inhibitors

Anti-PD1 therapy for LDH-A-deficient B16-F10 melanoma mice showed an increased anti-tumor immune response, compared with that of mice implanted with LDH-A-expressing tumors (Daneshmandi, Wegiel & Seth, 2019). In LDH-A-deficient tumors, the infiltration of NK cells and CD8(+) cytotoxic T cells increased, and the infiltration of Treg cells decreased, accompanied by a significant decrease in tumor growth (Daneshmandi, Wegiel & Seth, 2019). Thus, blocking LDH-A in tumors can improve the efficacy of ICIs, such as anti-PD1, which may be a new approach for cancer treatment (Daneshmandi, Wegiel & Seth, 2019). The acidity of the TME is one of the reasons for enhanced immune checkpoint expression. Diclofenac is a glycolytic inhibitor that reduces the acidification of the TME, and combined diclofenac and PD1/CTLA4 inhibitor therapy synergistically enhanced the anti-tumor immune function in a 4T1 mouse model (Lacroix et al., 2018). Oral bicarbonate neutralizes TME acidity and increases intratumoral pH. In a variety of tumor models, bicarbonate therapy in combination with anti-PD1 or CTLA4 Abs enhances the anti-tumor immune response (Pilon-Thomas et al., 2016). Chafe et al. demonstrated, in melanoma and breast cancer models, that CA9 inhibitors reduced TME acidity, and that CA9 inhibitors combined with PD1 and CTLA4 inhibitors can enhance the Th1 cell response and the immune response to immune checkpoint blockade (ICB), thus reducing tumor growth and metastasis (Chafe et al., 2019). CA9-targeted therapy combined with ICB may be an effective strategy for the treatment of hypoxic solid tumors. Currently, most clinical studies on glycolytic pathways or CA9 inhibitors have not yet published results *(*Table 2*)*. The published results of the phase III clinical trial NCT00087022 included 864 patients with renal clear cell carcinoma (RCC), randomly divided into CA9 inhibitor (Girentuximab) treatment group and placebo group, showed no significant difference in disease-free survival (DFS) and OS between the treatment group and the placebo group (Chamie et al., 2017). More clinical studies are needed to verify the efficacy and safety of glycolytic pathway or CA9 inhibitors.

#### EMT pathway inhibitors

EMT pathway inhibitors may improve the efficacy of immunotherapy. Noman et al. (2018) proposed that EMT inhibitors antagonize both PD1/PDL1 on T cells and CD47 on cancer cells during cancer treatment. This suggests that targeting the EMT pathway may enhance the blocking effect of PD1/PDL1 in patients with esophageal squamous cell carcinoma (Chen et al., 2018a), urothelial carcinoma (Mariathasan et al., 2018) and mesenchymal metastatic tumors (Noman et al., 2017), thus improving the antitumor immune response of T cells. The decrease in E-cadherin expression is an important characteristic of EMT. Thompson et al. (2020) found that down-regulation of E-cadherin expression negatively affected ICBs, suggesting that high expression of E-cadherin might be a marker for screening ICB-advantaged populations. The partial registered research projects for EMT inhibitors are listed in Table 2.

The up-regulation of CD47 expression in tumor cells is affected by the EMT. The up-regulation of CD47 inhibits the phagocytosis of macrophages, and inhibition of CD47 can inhibit tumor progression. Magrolimab is an anti-CD47 mAb that is part of an ongoing phase Ib clinical study (NCT03248479) (Sallman et al., 2019), in combination with azacitidine (DNMT inhibitor), in patients with myelodysplastic syndrome (MDS). Out of 18 evaluable patients, the ORR of combination therapy was as high as 100%, and 54% of these patients had a CR. Currently, phase III clinical trials (NCT04313881) are further evaluating the efficacy and safety of the combination of magrolimab and azacitidine. IBI322 is a bispecific antibody targeting PDL1 / CD47 simultaneously, which is part of a phase I clinical trial (NCT04328831) for the treatment of advanced malignant tumors, the results of which are highly anticipated.

## Conclusions

At present, most studies on the influence of hypoxia on immune checkpoint expression focus on PD1, PDL1, CTLA4, and CD47, while relatively few studies have examined the influence on other immune checkpoints, such as TIM3 and LAG3. These relatively unexplored immune checkpoints should be further studied as a matter of urgency, given the importance of immune checkpoints on tumor development. PD1/PDL1 inhibitors combined with CTLA4 inhibitors have yielded significant results in the treatment of solid tumors. Further studies have shown that the combination of HIF-1α inhibitors and Ado-inhibiting tumor immune microenvironment regulatory drugs with ICIs has demonstrated promising efficacy in both preclinical and phase I/II clinical studies. However, more basic and clinical studies are needed to verify the efficacy and safety of hypoxia pathway inhibition therapy.

With a deeper understanding of the TME, exploring the influence of tumor microenvironment hypoxia on the expression of immune checkpoints and the function of infiltrating immune cells can reveal the relationship between the hypoxic TME and immune escape, which is essential for the development of new drugs and the search for predictors of the efficacy of immunotherapy for treating malignant tumors. The combination of hypoxia pathway inhibitors and ICIs has shown preliminary efficacy in solid tumors, and might be a new method to combat drug resistance in tumor immunotherapy in the future. Therefore, the study of the relationship between the hypoxic microenvironment and immune escape might become a promising and attractive research direction in the future.

## Additional Information and Declarations

### Data Availability

The following information was supplied regarding data availability: This article is a Literature Review: there is no experimental data.
